# Longitudinal Displacement vs. Strain in Cardiac Amyloidosis: A Speckle Tracking Echocardiography Study

**DOI:** 10.3390/jcm15041544

**Published:** 2026-02-15

**Authors:** Marina Leitman, Vladimir Tyomkin, Shmuel Fuchs

**Affiliations:** 1 Department of Cardiology, Shamir Medical Center, Zerifin 70300, Israel; 2 Gray Faculty of Medical & Health Sciences, Tel Aviv University, Tel Aviv 6997801, Israel

**Keywords:** cardiac amyloidosis, echocardiography, strain, displacement, apical sparing

## Abstract

**Background**: Longitudinal strain is central to the echocardiographic diagnosis of cardiac amyloidosis, typically showing reduced global values with relative apical sparing. Longitudinal displacement—an absolute measure of myocardial motion—may provide complementary diagnostic and physiological information. **Methods**: We retrospectively studied 24 patients with cardiac amyloidosis and 24 age-, sex-, rhythm-, and ejection fraction–matched controls. Global and regional longitudinal strain and displacement were calculated. Diagnostic performance was evaluated using receiver-operating characteristic (ROC) analysis, and reproducibility was assessed using intraclass correlation coefficients (ICC), coefficient of variation (CV), and Bland–Altman analysis. **Results**: In amyloidosis, both global longitudinal strain (GLS) and global longitudinal displacement (GLD) were significantly reduced compared with controls (GLS: −10.2 ± 2.6% vs. −20.1 ± 2.4%, *p* < 0.0001; GLD: 6.6 ± 1.9 mm vs. 11.9 ± 1.4 mm, *p* < 0.0001). Amyloidosis was characterized by pronounced impairment of basal displacement (9.0 ± 4.4 vs. 17.0 ± 3.9 mm, *p* < 0.0001) and only modest reduction in absolute apical motion (3.0 ± 2.4 vs. 5.0 ± 2.3 mm, *p* < 0.0001), supporting the concept that apical sparing observed on strain reflects relative rather than absolute preservation of function. ROC analysis demonstrated strong discriminatory performance within this cohort for GLD (cutoff 8.8 mm), basal displacement (~13 mm), and GLS (absolute 15.8%), with areas under the curve approaching 1.0. GLD and GLS correlated with indices of diastolic burden and functional status (E/E′ and NYHA; |r| ≈ 0.32–0.41, all *p* ≤ 0.03). Reproducibility was good to excellent (ICC ≈ 0.84–0.89; CV 6–8%). **Conclusions**: Longitudinal displacement provides complementary and reproducible information alongside strain in cardiac amyloidosis. Combined assessment—reduced global or basal displacement together with reduced GLS and/or relative apical sparing—may refine the echocardiographic characterization of amyloid cardiomyopathy and link longitudinal mechanics to diastolic dysfunction and heart-failure burden.

## 1. Introduction

In recent years, substantial progress has been achieved in the understanding, diagnosis, and management of cardiac amyloidosis, driven by advances in noninvasive imaging techniques and increased clinical awareness. These developments have enabled earlier and more accurate identification of the disease, facilitating timely initiation of disease-modifying therapies—particularly in transthyretin cardiac amyloidosis (ATTR-CA) [1,2,3,4]. Contemporary diagnostic algorithms integrate echocardiographic findings, including characteristic longitudinal strain patterns, with cardiac magnetic resonance imaging and bone scintigraphy, underscoring the central role of myocardial mechanics within multimodality assessment frameworks [1,2,4,5].

Comprehensive transthoracic echocardiography remains a cornerstone in the evaluation of cardiac amyloidosis. In many cases of ATTR-CA, cardiac magnetic resonance imaging is not required when echocardiographic and clinical findings are strongly suggestive of the diagnosis [4]. Classic echocardiographic hallmarks include concentric left ventricular hypertrophy with preserved ejection fraction, subtle right ventricular dysfunction, biatrial enlargement, valvular and interatrial septal thickening, granular myocardial texture, small pericardial effusions, and diastolic dysfunction; low-gradient aortic stenosis is also frequently observed [4,5,6,7,8]. When interpreted in the appropriate clinical context, these findings may allow accurate diagnosis even in the absence of strain imaging [3].

In 2012, Phelan et al. first described the relative apical sparing pattern of longitudinal strain in cardiac amyloidosis, characterized by preserved apical strain in the setting of markedly reduced basal and mid-ventricular strain [9]. This observation established a reproducible echocardiographic signature of amyloid infiltration. Subsequent studies validated apical sparing as a diagnostic marker and proposed composite indices combining relative apical sparing with global longitudinal strain to enhance diagnostic accuracy [1,2,6,10].

However, apical sparing is not specific to amyloidosis. In normal hearts, apical strain physiologically exceeds basal strain, reflecting an intrinsic basal-to-apical gradient of longitudinal deformation [11]. Under pathological conditions, this gradient may become exaggerated or altered, resulting in a relative apical sparing pattern that must be interpreted in a clinical context [12]. Similar strain patterns have been described in severe aortic stenosis, where they correlate with the degree of left ventricular hypertrophy [13,14], as well as in hypertrophic cardiomyopathy [15], advanced chronic kidney disease [16], and other forms of myocardial hypertrophy [17]. These observations suggest that relative apical sparing may arise from remodeling-related gradients in myocardial mechanics rather than from amyloid infiltration alone.

Beyond strain-based indices, myocardial and annular motion parameters have long been used to assess longitudinal ventricular function. Tricuspid annular plane systolic excursion (TAPSE), introduced in the 1980s [18], remains a widely accepted measure of right ventricular systolic performance and has been incorporated into amyloidosis risk scores [1]. Similarly, mitral annular plane systolic excursion (MAPSE) was later proposed as a marker of left ventricular longitudinal function [19]. These measures emphasize the clinical relevance of absolute myocardial motion.

Longitudinal displacement—an absolute measure of myocardial excursion—may therefore provide complementary mechanistic and diagnostic information alongside strain [11,20]. In contrast to strain, which reflects relative deformation, displacement quantifies absolute motion and typically exhibits a reversed basal-to-apical gradient, with the greatest excursion in basal segments and minimal motion at the apex. Because strain and displacement capture distinct aspects of myocardial mechanics—relative shortening versus absolute motion—they may offer complementary insights into myocardial dysfunction in cardiac amyloidosis.

Accordingly, the present study aimed to evaluate left ventricular longitudinal displacement in patients with cardiac amyloidosis, to further elucidate the mechanical basis of relative apical sparing, and to assess the potential of displacement as a complementary diagnostic parameter to strain-based analysis.

## 2. Materials and Methods

### 2.1. Study Population

We retrospectively identified 24 consecutive patients with cardiac amyloidosis (Group 1) who underwent transthoracic echocardiography at our center between January and July 2025.

Cardiac amyloidosis was diagnosed according to established international criteria. Transthyretin cardiac amyloidosis was confirmed by typical echocardiographic findings in combination with positive bone-avid scintigraphy (Perugini grade ≥ 2) in the absence of monoclonal protein. Light-chain amyloidosis was diagnosed based on histological confirmation of amyloid deposition with immunohistochemical or immunofluorescent typing.

No patients in the present cohort had isolated atrial amyloidosis; all cases demonstrated ventricular involvement.

All studies were exported for offline analysis. Global and regional left ventricular longitudinal displacement and strain were derived from standard apical four-, two-, and three-chamber views as previously described [20]. For each patient, a three-plane composite plot of strain and displacement was generated to assess the spatial relationship between deformation and motion patterns.

A comparator group of 24 control subjects (Group 2), matched for age, sex, cardiac rhythm, and ejection fraction, underwent the same speckle-tracking workflow with calculation of longitudinal displacement and strain. Control subjects were selected to reflect a real-world elderly echocardiography population in whom cardiac amyloidosis is commonly considered, rather than healthy volunteers. Accordingly, common comorbidities were not excluded.

### 2.2. Echocardiographic Acquisition and Measurements

All examinations were performed using a Vivid E95 ultrasound system (GE Healthcare, Horten, Norway) equipped with a 1.7–4.0 MHz transducer. Frame rates for speckle tracking were optimized and maintained at ≥50 frames/s (typically 50–90 frames/s).

Comprehensive transthoracic imaging was obtained according to current guideline recommendations for chamber quantification [20], including parasternal long- and short-axis views (basal, mid-ventricular, and apical levels) and apical four-, two-, and three-chamber views.

Diastolic function was assessed according to contemporary recommendations [21], including mitral E and A velocities, E/A ratio, E-wave deceleration time, tissue Doppler E′ velocities at the septal and lateral annulus, and calculation of E/E′.

Left atrial volume was calculated using the biplane area-length method:

LA volume = (8/3π) × (A4ch × A2ch)/L

where A4ch and A2ch represent left atrium areas obtained from the apical four-chamber and two-chamber views, respectively, and L is the shortest atrial long-axis length. Left atrial volume index (LAVi) was obtained by indexing to body surface area (BSA).

Left ventricular mass (LVM) was calculated using the Devereux formula:

$$0.8*1.04*\left[{\left(IVS+PW+LVID\right)}^{3}-{LVID}^{3}\right]+0.6g$$

where IVS is the interventricular septal thickness, PW is the posterior wall thickness, and LVID is the left ventricular internal diameter at end-diastole. LVM was indexed to BSA (LVMi).

Relative wall thickness (RWT) was calculated as:

RWT = (2 ∗ PW)/LVEDD

where LVEDD is left ventricular end-diastolic diameter.

### 2.3. Speckle-Tracking Analysis (Strain and Displacement)

All studies were analyzed offline using EchoPAC software (Version 206, GE Healthcare). Endocardial borders were traced using automated tracking with manual adjustment when necessary. The 17-segment AHA model was applied. End-systole was defined by aortic valve closure in the apical long-axis view and verified using Doppler in the apical five-chamber view.

### 2.4. Longitudinal Strain (Global and Regional)

Peak systolic longitudinal strain was measured in each segment, and global longitudinal strain (GLS) was calculated as the average of the three apical views (four-, two-, and three-chamber). Regional strain values were calculated as mean values for basal, mid-ventricular, and apical rings. GLS is reported as a negative value (more negative values indicate greater shortening).

### 2.5. Longitudinal Displacement (Global and Regional)

Longitudinal displacement was derived from the same apical views. Global longitudinal displacement (GLD) was calculated as the mean displacement of all segments. Regional displacement was averaged for basal, mid-ventricular, and apical rings, as previously described [11], and is reported in millimeters.

### 2.6. Reproducibility

A reproducibility substudy was conducted in 20 patients (10 per group), including three examinations with atrial fibrillation per group and two women in each group. Global (primary endpoint) and regional (secondary endpoints) longitudinal strain and displacement were reanalyzed to assess inter-observer (ML vs. VT) and intra-observer (ML repeated analysis) variability.

Reproducibility metrics included: intraclass correlation coefficient [ICC(2, 1)] with 95% confidence intervals; coefficient of variation (CV%); Bland–Altman mean bias ± 95% limits of agreement; standard error of measurement (SEM); and repeatability coefficient (RC).

### 2.7. Statistical Analysis

Continuous variables are presented as mean ± SD and categorical variables as n (%). Normality was assessed using the Shapiro–Wilk test and visual inspection of Q–Q plots. Between-group comparisons were performed using independent-samples *t* tests or Mann–Whitney U tests, as appropriate. Categorical variables were compared using χ^2^ or Fisher’s exact tests. Associations were evaluated using Pearson (linear) and Spearman (rank) correlation coefficients. Diagnostic performance was assessed using receiver operating characteristic (ROC) analysis. Areas under the curve (AUCs) with 95% confidence intervals were calculated using the DeLong method. Optimal thresholds were determined using the Youden index. Statistical significance was defined as a two-sided *p*-value < 0.05. All analyses were performed using IBM SPSS Statistics version 28.0 (IBM, Armonk, NY, USA).

### 2.8. Ethics

The study was approved by the Helsinki Ethics Committee of Shamir (Assaf Harofeh) Medical Center (approval No. 0122-25-ASF(V3), 29 July 2025). All echocardiographic data were fully de-identified prior to analysis. The study was conducted in accordance with institutional policies and the principles of the Declaration of Helsinki. Given the retrospective and anonymized study design, the requirement for written informed consent was waived.

## 3. Results

### 3.1. General Characteristics

Baseline characteristics are summarized in Table 1. Mean age was 84.8 ± 5 years in the amyloidosis group and 82.6 ± 8 years in controls (*p* = 0.30). Both groups included 20 males (83.3%). The prevalence of major comorbidities was similar between groups. Among patients with amyloidosis, 21 had transthyretin (ATTR) and 3 had light-chain (AL) disease. Atrial fibrillation during echocardiography was present in 10 patients (41.7%) in each group. NYHA functional class tended to be higher in the amyloidosis group (2.8 ± 0.5 vs. 2.3 ± 0.8; *p* = 0.05).

### 3.2. Echocardiographic Characteristics

Echocardiographic characteristics are summarized in Table 2. Left ventricular ejection fraction did not differ significantly between groups. However, patients with amyloidosis exhibited more pronounced concentric hypertrophy, with higher left ventricular mass index and greater septal and posterior wall thickness. Left ventricular end-diastolic diameter was smaller in amyloidosis, whereas end-systolic diameter was similar.

Indices of diastolic function demonstrated significantly higher E/E′ values in the amyloidosis group, consistent with elevated filling pressures. Tricuspid annular peak systolic velocity was lower in amyloidosis, while pulmonary artery pressure and left atrial volume index were comparable between groups.

Both global longitudinal strain and global longitudinal displacement were significantly reduced in amyloidosis compared with controls.

### 3.3. Regional Left Ventricular Displacement and Strain

Regional longitudinal displacement and strain were reduced across basal, mid-ventricular, and apical levels in patients with amyloidosis compared with controls (Table 3).

**Figure 1:**
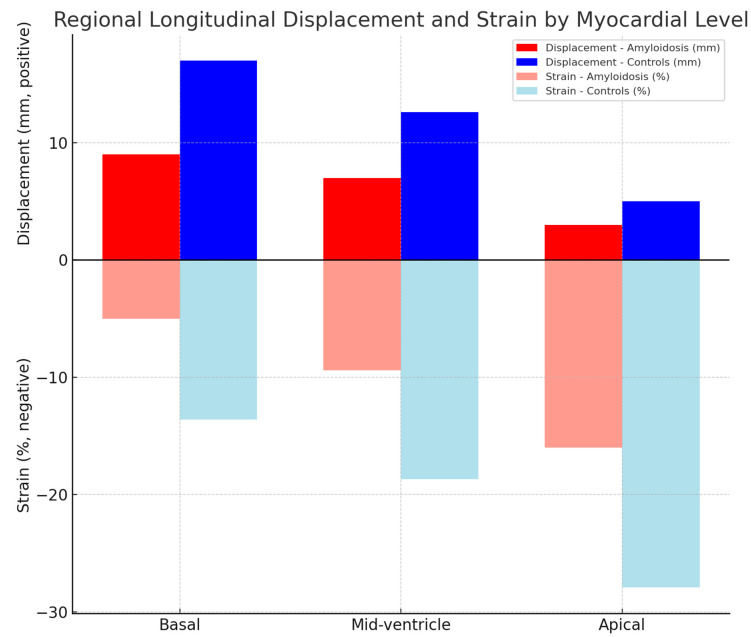
Regional displacement and strain by myocardial level. Bar chart illustrating basal, mid, and apical displacement (mm, positive values) and strain (%, negative values) in patients with amyloidosis (red/pink) compared with controls (blue/light blue). Both displacement and strain were significantly lower in amyloidosis across all myocardial levels, with relative preservation at the apex consistent with the apical sparing pattern.

At each myocardial level, both displacement and strain were lower in amyloidosis (Table 4; Figure 1). Basal displacement was markedly reduced (9.0 ± 4.4 vs. 17.0 ± 3.9 mm; *p* < 0.0001), whereas apical displacement showed a smaller absolute reduction (3.0 ± 2.4 vs. 5.0 ± 2.3 mm; *p* < 0.0001). These findings support that the strain-based apical sparing pattern reflects relative rather than absolute preservation of myocardial function.

In both groups, basal displacement exceeded mid-ventricular displacement, and mid-ventricular displacement exceeded apical displacement, demonstrating the expected reversed basal-to-apical displacement gradient (Table 5). In contrast, longitudinal strain exhibited the physiological basal-to-apical gradient, with more negative values toward the apex. In the amyloidosis group, these between-level differences were more pronounced, resulting in the characteristic relative apical sparing pattern.

**Figure 2:**
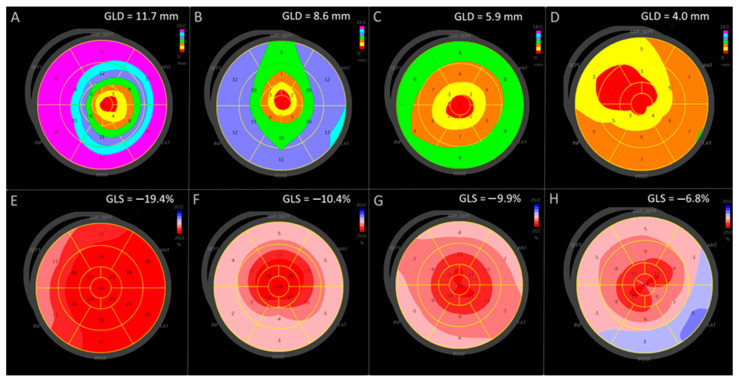
Progressive impairment of longitudinal displacement and strain in cardiac amyloidosis. Panels (**A**–**D**) show longitudinal displacement (mm); panels (**E**–**H**) show longitudinal strain (%), all derived from apical 4-, 2-, and 3-chamber views. (**A**,**E**) are from a control subject; (**B**–**D**,**F**–**H**) illustrate cardiac amyloidosis with increasing mechanical impairment. Color bars at right indicate magnitude (displacement row: higher values toward lilac; strain row: more negative strain toward darker red). Numbers denote segmental values. Scales differ between rows (displacement 0–18 mm; strain 0 to –20%); for strain, more negative values indicate greater shortening. Abbreviations: ANT = anterior, SEPT = septal, LAT = lateral, POST = posterior. In the control map (**A**), a prominent outer lilac ring denotes brisk basal displacement. With amyloidosis progression, the displacement maps show a stepwise loss of basal motion: first, the lilac outer band disappears (**B**), then the blue bands (**C**), and subsequently the green band (**D**), leaving only low-amplitude motion centered near the apex. The strain maps mirror this deterioration: compared with the control (**E**), strain is progressively reduced in amyloidosis (**F**–**G**), and in advanced disease, the zone of reduced absolute motion extends toward the apex, leading to further attenuation of apical strain (**H**).

Figure 2 illustrates graded impairment of myocardial mechanics in cardiac amyloidosis. Compared with the control maps, both longitudinal displacement (A–D) and longitudinal strain (E–H) are progressively reduced. The characteristic outer lilac band on the displacement bull’s-eye—representing the largest absolute motion in basal segments in normal myocardium—is attenuated or absent in amyloidosis, consistent with pronounced basal hypokinesia and a parallel reduction in global myocardial deformation.

### 3.4. Correlations with Diastolic Function and NYHA Class

**Figure 3:**
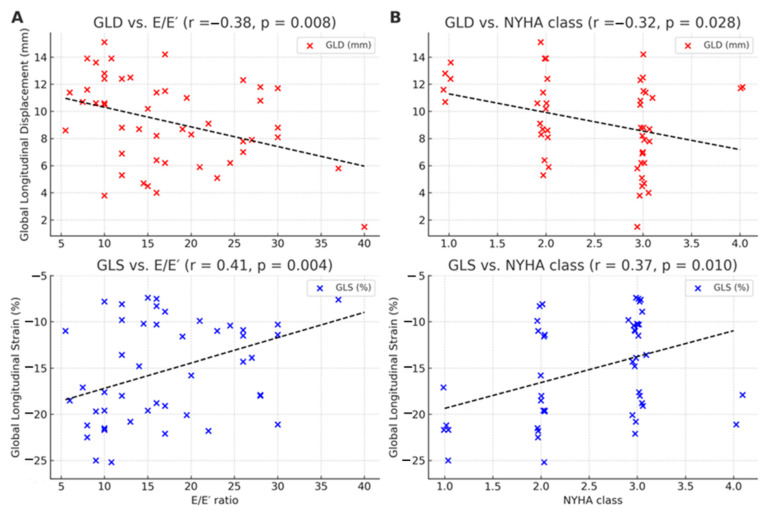
Correlation of global longitudinal displacement (GLD) and global longitudinal strain (GLS) with diastolic function (E/E′) and NYHA class. (**A**) GLD (**top**) shows a significant inverse correlation with E/E′ (r = −0.38, *p* = 0.008). GLS (**bottom**, signed values) shows a significant positive correlation with E/E′ (r = +0.41, *p* = 0.004), indicating that as filling pressures rise, strain magnitude becomes less negative (i.e., worsens). (**B**) GLD (**top**) is modestly inversely correlated with NYHA class (r = −0.32, *p* = 0.028). GLS (**bottom**, signed values) is modestly positively correlated with NYHA class (r = +0.37, *p* = 0.010), reflecting lower strain magnitude with worse functional status. Points are individual subjects; black dashed lines are least-squares fits. Together, these associations support the physiological link between impaired longitudinal mechanics and elevated filling pressures/clinical severity in cardiac amyloidosis.

Global longitudinal displacement and global longitudinal strain (analyzed as absolute values) correlated modestly with NYHA functional class (GLD: r = −0.32; |GLS|: r = −0.37) and with E/E′ (GLD: r = −0.38; |GLS|: r = −0.41; all *p* ≤ 0.017), indicating that impaired longitudinal mechanics are associated with higher filling pressures and greater clinical severity (Table 6; Figure 3). No significant correlations were observed with left atrial volume index or pulmonary artery pressure.

### 3.5. Diagnostic Performance of Strain and Displacement (ROC Analysis)

**Figure 4:**
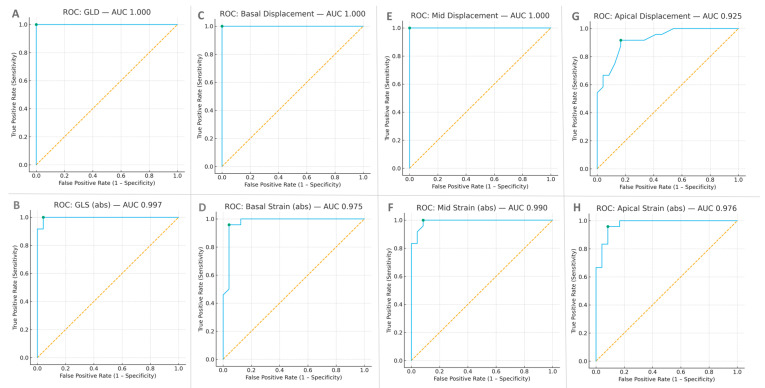
Diagnostic performance of displacement and strain by ROC analysis. Panels show receiver-operating characteristic (ROC) curves for global displacement (**A**), global longitudinal strain (**B**), basal displacement (**C**), basal strain (**D**), mid displacement (**E**), mid strain (**F**), apical displacement (**G**), and apical strain (**H**). Green markers indicate optimal diagnostic thresholds (Youden index). ROC curves for GLD and basal displacement nearly overlap in the upper-left corner, indicating high diagnostic accuracy. AUC = area under the curve. The dashed diagonal line represents the reference line of no discrimination (AUC = 0.5).

Receiver operating characteristic (ROC) analysis was performed for global and regional indices of longitudinal mechanics (Table 7; Figure 4). In this cohort (n = 48), global longitudinal displacement, global longitudinal strain, and basal displacement demonstrated strong discriminatory performance for differentiating amyloidosis from controls.

The optimal global longitudinal displacement cutoff of 8.8 mm achieved complete separation of groups in this dataset. Global longitudinal strain (threshold 15.8%, absolute value) and basal displacement yielded similarly high AUC values. Mid-ventricular indices also showed strong performance, whereas apical measures demonstrated comparatively lower discrimination, consistent with smaller intrinsic apical excursion and greater tracking variability.

Importantly, these diagnostic thresholds should be interpreted cautiously, given the modest sample size and single-center design.

As illustrated in Figure 4, the ROC curves for global longitudinal displacement and basal displacement cluster in the upper-left region of the plot and show substantial overlap, indicating strong classification performance within this cohort. These findings suggest that displacement-based indices provide diagnostic information that is complementary to strain. While global longitudinal strain demonstrated its expected discriminatory capacity, displacement measures—particularly global and basal displacement—offer an absolute motion-based perspective that complements the relative deformation captured by strain. Together, these parameters characterize the echocardiographic profile of cardiac amyloidosis, combining reduced global strain with relative apical sparing and a pronounced reduction in basal longitudinal displacement.

### 3.6. Reproducibility

**Figure 5:**
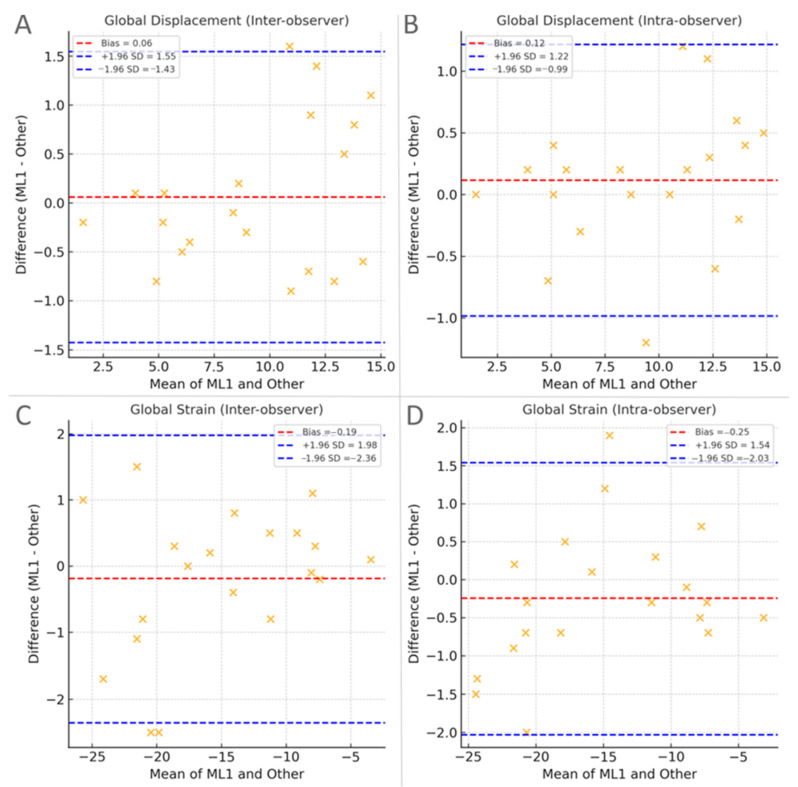
Bland–Altman plots for reproducibility of global measurements. (**A**) Reproducibility of global displacement, Inter-observer agreement (ML vs. VT). (**B**) Reproducibility of global displacement, Intra-observer agreement (ML). (**C**) Reproducibility of global strain, Inter-observer agreement (ML vs. VT). (**D**) Reproducibility of global strain, Intra-observer agreement (ML). Each plot shows the mean of the two measurements on the *x*-axis and their difference on the *y*-axis. The red dashed line indicates the bias (mean difference), and the blue dashed lines represent the 95% limits of agreement (bias ± 1.96 SD).

Inter- and intra-observer reproducibility was high for both global and regional measures (Table 8; Figure 5). For global indices, inter-observer ICCs were 0.888 for strain and 0.845 for displacement, and intra-observer ICCs were 0.883 and 0.837, respectively. Coefficients of variation were low (6–8%), and mean biases were small with narrow limits of agreement.

Basal and mid-ventricular measurements demonstrated slightly higher reproducibility than apical indices, which demonstrated modestly wider limits of agreement, consistent with lower motion amplitude and tracking variability at the apex.

Overall, these findings indicate that both longitudinal strain and displacement provide reproducible quantitative indices of left ventricular longitudinal mechanics, supporting the feasibility of displacement analysis in both clinical and research settings.

## 4. Discussion

### 4.1. Principal Findings

In this age-, sex-, rhythm-, and ejection fraction-matched cohort, global longitudinal displacement, global longitudinal strain, and basal displacement demonstrated strong diagnostic discrimination between cardiac amyloidosis and controls. Both GLD and GLS correlated with E/E′ and NYHA functional class, linking impaired longitudinal mechanics to elevated filling pressures and clinical heart-failure burden. Reproducibility analyses showed good agreement for global and regional measures, supporting longitudinal displacement as a feasible and complementary parameter alongside strain in routine echocardiographic assessment.

### 4.2. Mechanism of Apical Sparing

**Figure 6:**
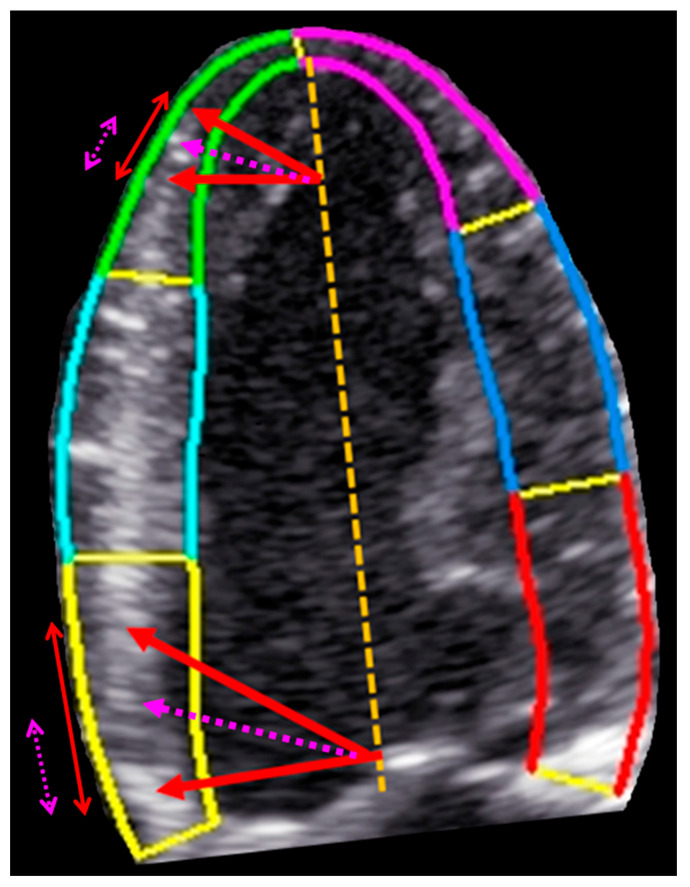
Mechanistic explanation of apical sparing using absolute displacement versus relative strain.

The left ventricle exhibits geometric heterogeneity along its long axis. Basal segments traverse a larger radius and longer arc during systole, resulting in greater absolute longitudinal displacement. In contrast, the apex—characterized by sharper curvature and geometric tethering—demonstrates smaller absolute motion. This geometric framework provides a mechanistic basis for relative apical sparing and is illustrated schematically in Figure 6.

Schematic apical four-chamber view with color-coded endocardial contours. Red double-headed arrows represent absolute longitudinal displacement in a normal subject (arrow length proportional to displacement magnitude), while lilac dashed double-headed arrows represent longitudinal displacement in cardiac amyloidosis. The yellow dashed line indicates the left ventricular long axis.

In normal ventricular mechanics (red arrows), basal segments demonstrate the greatest absolute longitudinal displacement during systole, whereas the apex exhibits smaller absolute motion. This occurs because the radius of curvature at the apex is smaller than at the base, limiting the magnitude of absolute longitudinal movement despite preserved contractility. However, because apical myocardial shortening occurs over a shorter baseline length, relative deformation (strain) is higher at the apex, producing the normal basal-to-apical strain gradient.

In cardiac amyloidosis (lilac dashed arrows), amyloid infiltration and myocardial stiffening predominantly impair basal and mid-ventricular mechanics, resulting in a marked reduction of absolute displacement and strain in these regions. Apical segments, which are relatively less affected, maintain comparatively higher strain despite limited absolute motion. This disproportionate basal impairment leads to the characteristic pattern of relative apical sparing.

Together, these patterns illustrate how ventricular geometry and differential myocardial involvement explain why displacement and strain provide complementary diagnostic information in cardiac amyloidosis.

Conceptually, longitudinal strain may be expressed as ε ≈ ΔL/L_0_, where ΔL represents absolute systolic displacement and L_0_ the baseline segment length. At the apex, a short L_0_ means that even modest absolute motion produces relatively large strain, whereas at the base, greater displacement distributed over a longer arc results in smaller relative deformation.

Accordingly, in normal hearts, displacement follows a reversed basal-to-apical gradient (basal > mid > apical), while strain exhibits the physiological basal-to-apical gradient (more negative toward the apex) [22]. In amyloidosis, extracellular amyloid deposition predominates in the basal and mid segments, reducing both displacement and strain. The apex is relatively less affected, so apical strain appears comparatively preserved despite reduced absolute motion—yielding the characteristic relative apical sparing pattern [23,24,25,26].

Importantly, apical sparing should not be interpreted as preserved apical function. In our cohort, apical displacement was significantly reduced in amyloidosis compared with controls, albeit less than at basal levels. Thus, the strain pattern reflects disproportionate basal impairment rather than true apical preservation.

Emerging histopathologic data suggest that amyloid deposition follows a continuum rather than a uniform basal-to-apical gradient [27]. Diffuse low-burden, predominantly basal, and diffuse high-burden phenotypes likely represent stages of disease progression. Displacement patterns may therefore evolve with advancing infiltration and remodeling. Evaluating both relative deformation and absolute motion may enhance understanding of this disease spectrum (Figure 2).

### 4.3. Added Value of Displacement Alongside Strain

Strain remains the reference deformation index in cardiac amyloidosis and underpins apical sparing-based diagnostic approaches [1,2,3,4,5,6,7,9]. However, strain reflects relative deformation and does not quantify absolute myocardial excursion.

Displacement provides motion-based information that complements strain-derived metrics. In our cohort, global and basal displacement performed comparably to GLS in differentiating amyloidosis from controls. Displacement captures basal hypokinesia and reduced excursion typical of amyloid infiltration, while strain preserves the recognizable apical sparing signature.

From a clinical standpoint, displacement may provide added insight when GLS values are borderline, speckle-tracking quality is suboptimal, or marked wall thickening limits deformation analysis despite visually appreciable motion. In such cases, displacement may refine interpretation rather than replace strain.

Markedly reduced basal and mid-ventricular motion may contribute to reduced stroke volume and cardiac output, potentially explaining the frequent association between amyloidosis and low-flow, low-gradient severe aortic stenosis [8,28].

Although displacement and strain showed strong concordance, this reflects shared physiological underpinnings rather than redundancy. Strain expresses relative shortening, whereas displacement quantifies absolute motion. In amyloidosis, this distinction clarifies that apparent apical preservation on strain imaging does not imply preserved absolute contraction.

Atrial fibrillation may influence longitudinal displacement by altering loading conditions, cycle-length variability, and atrioventricular coupling. Irregular RR intervals and loss of atrial contraction can increase measurement variability. Although displacement reflects absolute excursion, rhythm-related hemodynamic fluctuations may still modulate longitudinal motion. In this study, groups were rhythm-matched; however, the modest sample size precluded formal subgroup analyses. Rhythm-specific effects on displacement, therefore, warrant further investigation in larger cohorts.

### 4.4. Relationship to Diastolic Function and Clinical Status

Both GLD and GLS correlated with E/E′ and NYHA classes, reinforcing the link between impaired longitudinal mechanics, elevated filling pressures, and clinical severity. These associations support the concept that longitudinal dysfunction in amyloidosis reflects both structural infiltration and functional compromise.

### 4.5. Diagnostic Performance and Thresholds

ROC analysis demonstrated strong discrimination for global and basal displacement as well as GLS. However, given the modest sample size and single-center design, these thresholds should be interpreted cautiously. The very high AUC values observed may reflect optimistic bias related to the small sample size, and internal derivation of thresholds should be considered hypothesis-generating pending validation in larger, independent cohorts. Apical indices demonstrated comparatively lower performance, consistent with smaller intrinsic excursion and greater tracking variability at the apex.

### 4.6. Reproducibility and Feasibility

Global displacement and strain showed good inter- and intra-observer reproducibility. Regional basal and mid-level measures were slightly more reproducible than apical indices, likely reflecting greater motion amplitude and more stable tracking. These findings support the technical feasibility of displacement analysis as an adjunct to strain.

### 4.7. Clinical Implications

Displacement and strain offer complementary perspectives:

Deformation lens (GLS + apical sparing): captures relative shortening patterns.

Motion lens (GLD + basal displacement): quantifies loss of basal excursion and attenuation of the reversed displacement gradient.

When both approaches converge—reduced GLS with apical sparing and reduced basal displacement—diagnostic confidence increases. In equivocal cases or phenotypic mimics, the displacement profile may assist in clinical decision-making and triage for further evaluation.

## 5. Limitations

This single-center study’s modest sample size and retrospective design may limit generalizability and introduce selection bias. Vendor-specific implementation (GE EchoPAC) may restrict external applicability, and cross-vendor validation of displacement algorithms is warranted. The very high AUC values should be interpreted cautiously, as small datasets are vulnerable to overfitting. Identified thresholds require prospective validation before clinical adoption.

The study did not include dedicated comparator groups with specific hypertrophic phenotypes such as hypertensive heart disease or aortic stenosis. Subtype-specific analyses were not feasible due to the small number of AL cases. Rhythm-related effects on displacement, particularly in atrial fibrillation, warrant further dedicated investigation.

## 6. Conclusions

In cardiac amyloidosis, longitudinal displacement provides reproducible and complementary information alongside strain. Combined evaluation refines the interpretation of the apical sparing phenomenon and enhances the mechanistic understanding of myocardial dysfunction in amyloid cardiomyopathy. Larger prospective studies are required to validate diagnostic thresholds and determine incremental clinical value.

## Figures and Tables

**Table 1:** General characteristics of patients with cardiac amyloidosis (Group 1) and controls (Group 2).

Variable	Group 1	Group 2	*p*-Value
Patients, n	24	24	1.0
Male sex, n (%)	20 (83.3)	20 (83.3)	1.0
Age, years	84.8 ± 5	82.6 ± 8	0.3
Atrial Fibrillation during echocardiography, n (%)	10 (41.7)	10 (41.7)	1.0
Weight, kg	71.1 ± 11.1	77.4 ± 14.6	0.1
Height, cm	166.3 ± 9.2	169.7 ± 7.6	0.2
BMI, kg/m^2^	25.5 ± 3	26.8 ± 4.4	0.3
BSA, m^2^	1.8 ± 0.2	1.9 ± 0.2	0.1
Hypertension, n (%)	17 (70.8)	20 (83.3)	0.4
Diabetes mellitus, n (%)	9 (37.5)	9 (37.5)	1.0
Chronic kidney disease, n (%)	15 (62.5)	10 (41.7)	0.9
Ischemic heart disease, n (%)	6 (25.0)	6 (25.0)	1.0
Chronic pulmonary disease, n (%)	6 (25.0)	9 (37.5)	0.6
History of atrial fibrillation, n (%)	16 (66.7)	14 (58.3)	0.8
Malignancy, n (%)	5 (20.8)	9 (37.5)	0.4
NYHA, functional class	2.8 ± 0.5	2.3 ± 0.8	0.05

**Table 2:** Echocardiographic characteristics of patients with cardiac amyloidosis (Group 1) and controls (Group 2).

Variable	Group 1	Group 2	*p*-Value
Heart rate, beats/min	72 ± 12.6	75.4 ± 14.0	0.4
Ejection fraction, %	53.8 ± 7.3	55.3 ± 4.0	0.37
Left ventricular mass index, g/m^2^	145.0 ± 30.1	115.2 ± 63.8	<0.05
Regional wall thickness	0.66 ± 0.13	0.47 ± 0.06	<0.0001
Interventricular thickness, cm	1.7 ± 0.3	1.4 ± 0.3	<0.0001
Posterior wall thickness, cm	1.3 ± 0.2	1.1 ± 0.2	<0.0001
Left ventricle end diastolic diameter, cm	4.2 ± 0.5	4.6 ± 0.7	0.03
Left ventricle end systolic diameter, cm	2.7 ± 0.5	2.8 ± 0.6	0.6
E/E′	20.1 ± 8.4	14.7 ± 7.1	0.02
E deceleration, msec	189.2 ± 53.5	190.9 ± 65.5	0.9
Left atrial volume index, mL/m^2^	54.9 ± 15.8	53.8 ± 25.9	0.9
Tricuspid annulus peak systolic velocity, cm/s	1.5 ± 0.4	2.1 ± 0.4	<0.0001
Pulmonary artery pressure, mm Hg	44.6 ± 15.3	41.7 ± 11.5	0.5
Global longitudinal strain, %	−10.2 ± 2.6	−20.1 ± 2.4	<0.0001
Global longitudinal displacement, mm	6.6 ± 1.9	11.9 ± 1.4	<0.0001

Abbreviations: LV = left ventricle; TAPSE = tricuspid annular plane systolic excursion; E/E′ = ratio of mitral inflow velocity to annular early diastolic velocity.

**Table 3:** Regional displacement and strain values in patients with cardiac amyloidosis (Group 1) and controls (Group 2).

Segment	Variable	Group 1	Group 2	*p*-Value	Group 1	Group 2	*p*-Value
		Displacement			Strain		
Basal segments	Antero-septal	6.6 ± 4.1	14.1 ± 3.4	<0.0001	−6.8 ± 4.0	−15.7 ± 3.6	<0.0001
	Septal	6.8 ± 5.4	18.2 ± 4.3	<0.0001	−4.3 ± 2.6	−10.9 ± 3.8	<0.0001
	Inferior	10.2 ± 3.0	18.9 ± 4.0	<0.0001	−5.6 ± 3.1	−13.2 ± 5.1	<0.0001
	Posterior	10.8 ± 4.2	18.3 ± 2.8	<0.001	−2.8 ± 4.6	−14.1 ± 6.2	<0.0001
	Lateral	9.7 ± 3.8	17.0 ± 3.8	<0.0001	−4.4 ± 6.0	−14.1 ± 6.4	<0.0001
	Anterior	10.1 ± 3.8	15.6 ± 2.9	0.001	−6.2 ± 3.7	−13.8 ± 5.5	<0.0001
Mid-ventricular segments	Antero-septal	4.4 ± 3.5	9.9 ± 3.4	<0.0001	−11.3 ± 4.8	−21.7 ± 3.3	<0.0001
	Septal	5.2 ± 5.0	14.4 ± 4.3	<0.0001	−8.4 ± 3.1	−18.0 ± 3.2	<0.0001
	Inferior	8.0 ± 2.4	14.6 ± 3.1	<0.0001	−9.6 ± 3.5	−18.3 ± 4.3	<0.0001
	Posterior	9.1 ± 4.2	13.3 ± 2.6	<0.001	−7.6 ± 3.5	−18.2 ± 3.8	<0.0001
	Lateral	7.8 ± 3.4	12.1 ± 3.3	<0.0001	−9.2 ± 4.8	−17.7 ± 6.5	<0.0001
	Anterior	7.6 ± 3.9	11.1 ± 2.8	<0.001	−10.6 ± 3.3	−18.3 ± 5.5	<0.0001
Apical segments	Septal	1.7 ± 2.4	4.9 ± 3.3	<0.001	−15.6 ± 5.2	−29.6 ± 3.7	<0.0001
	Inferior	3.7 ± 1.4	6.0 ± 1.8	<0.0001	−16.6 ± 5.1	−28.3 ± 3.7	<0.0001
	Lateral	4.1 ± 2.9	4.9 ± 2.3	0.3	−15.1 ± 4.4	−27.2 ± 4.1	<0.0001
	Anterior	2.4 ± 2.5	4.0 ± 1.9	<0.02	−16.8 ± 4.9	−28.5 ± 4.3	<0.0001
	Apex	3.1 ± 1.4	5.0 ± 0.8	<0.0001	−15.8 ± 4.4	−25.9 ± 11.8	<0.001

**Table 4:** Displacement and strain in basal, mid-ventricular, and apical segments in patients with cardiac amyloidosis (Group 1) and controls (Group 2).

Segment	Displacement (mm)			Strain (%)		
	Group 1	Group 2	*p* Value	Group 1	Group 2	*p* Value
Basal	9.0 ± 4.4	17.0 ± 3.9	<0.0001	−5.0 ± 4.3	−13.6 ± 5.4	<0.0001
Mid-ventricle	7.0 ± 4.2	12.6 ± 3.7	<0.0001	−9.4 ± 4.1	−18.7 ± 4.8	<0.0001
Apical	3.0 ± 2.4	5.0 ± 2.3	<0.0001	−16.0 ± 4.8	−27.9 ± 6.5	<0.0001

**Table 5:** Significance of displacement and strain across left ventricular levels in patients with cardiac amyloidosis (Group 1) and controls (Group 2).

Segments	Basal	Mid-Ventricle	Apex	P1	P2
Displacement Group 1	9.0 ± 4.4	7.0 ± 4.2	3.0 ± 2.4	<0.0001	<0.0001
Displacement Group 2	17.0 ± 3.9	12.6 ± 3.7	5.0 ± 2.3	<0.0001	<0.0001
Strain Group 1	−5.0 ± 4.3	−9.4 ± 4.1	−16.0 ± 4.8	<0.0001	<0.0001
Strain Group 2	−13.6 ± 5.4	−18.7 ± 4.8	−27.9 ± 6.5	<0.0001	<0.0001

P1 = significance between basal and mid-ventricular segments; P2 = significance between mid-ventricular and apical segments.

**Table 6:** Correlations of global longitudinal displacement (GLD) and global longitudinal strain (GLS) with clinical and echocardiographic parameters.

Metric	Variable	Pearson r	*p*-Value	Spearman ρ	*p*-Value
GLD	NYHA	–0.33	0.028	–0.31	0.034
GLS	NYHA	0.37	0.010	0.39	0.007
GLD	E/E′	–0.38	0.008	–0.34	0.017
GLS	E/E′	–0.41	0.004	–0.36	0.013
GLD	LAVi	–0.07	0.62	–0.16	0.27
GLS	LAVi	–0.07	0.63	–0.15	0.3
GLD	PAP	–0.14	0.36	–0.18	0.21
GLS	PAP	–0.11	0.49	–0.14	0.38

GLD—Global longitudinal displacement, GLS—global longitudinal strain.

**Table 7:** Receiver Operating Characteristic (ROC) analysis in patients with amyloidosis and in controls.

Measure	Threshold	Sensitivity	Specificity	Accuracy	AUC (95% CI)	n Amyloidosis	n Controls	n Total
GLD, mm	8.8	100.0%	100.0%	100.0%	1.000–1.000	24	24	48
GLS, %, abs	15.8	100.0%	96.2%	98.0%	0.983–1.000	24	24	48
Basal Displacement, mm	13.2	100.0%	100.0%	100.0%	1.000–1.000	24	24	48
Basal Strain, %, abs	10	95.8%	96.2%	96.0%	0.920–1.000	24	24	48
Mid Displacement, mm	9.5	100.0%	100.0%	100.0%	1.000–1.000	24	24	48
Mid Strain, %, abs	14.2	100.0%	92.3%	96.0%	0.967–1.000	24	24	48
Apical Displacement, mm	4.2	91.7%	84.6%	88.0%	0.839–0.984	24	24	48
Apical Strain, %, abs	23.2	95.8%	92.3%	94.0%	0.932–1.000	24	24	48

GLD—Global longitudinal displacement, GLS—global longitudinal strain, AUC—area under the curve, and CI—confidence interval.

**Table 8:** Reproducibility of Strain and Displacement.

Parameter	Type	ICC	CV%	Bias ± LoA	SEM/RC
Global Strain, %	Inter-observer	0.888	7	−0.19 (−2.36 to +1.98)	0.78/3.06
Global Displacement, mm	Inter-observer	0.845	8	+0.06 (−1.43 to +1.55)	0.54/2.10
Global Strain, %	Intra-observer	0.883	6	−0.25 (−2.03 to +1.54)	0.64/2.52
Global Displacement, mm	Intra-observer	0.837	6	+0.12 (−0.99 to +1.22)	0.40/1.56
Basal Strain, %	Inter-observer	0.89	7	−0.19 (−2.36 to +1.98)	0.78/3.06
Mid Strain, %	Inter-observer	0.89	7	−0.02 (−1.95 to +1.91)	0.70/2.74
Apex Strain, %	Inter-observer	0.87	8	−0.32 (−2.71 to +2.07)	0.83/3.24
Basal Displacement, mm	Inter-observer	0.88	7	+0.08 (−1.69 to +1.84)	0.64/2.50
Mid Displacement, mm	Inter-observer	0.83	8	+0.15 (−1.47 to +1.77)	0.58/2.29
Apex Displacement, mm	Inter-observer	0.78	11	+0.14 (−0.68 to +0.96)	0.29/1.15
Basal Strain, %	Intra-observer	0.88	6	−0.25 (−2.03 to +1.54)	0.64/2.52
Mid Strain, %	Intra-observer	0.89	7	−0.31 (−2.08 to +1.47)	0.64/2.52
Apex Strain, %	Intra-observer	0.86	7	−0.46 (−2.84 to +1.92)	0.85/3.32
Basal Displacement, mm	Intra-observer	0.88	7	+0.08 (−1.69 to +1.84)	0.64/2.50
Mid Displacement, mm	Intra-observer	0.83	9	+0.17 (−1.60 to +1.94)	0.64/2.51
Apex Displacement, mm	Intra-observer	0.79	10	+0.12 (−0.66 to +0.90)	0.27/1.08

ICC = intraclass correlation coefficient; CV = coefficient of variation; SEM = standard error of measurement; RC = repeatability coefficient; LoA = limits of agreement.

## Data Availability

Data are available from the corresponding author upon reasonable request.
